# YouTube as a Source of Patient Information on Oral Manifestations of COVID-19: A Cross-Sectional Evaluation of Its Utility, Dependability, and Content

**DOI:** 10.7759/cureus.42885

**Published:** 2023-08-03

**Authors:** Salwa A Aldahlawi, Lujain Homeida

**Affiliations:** 1 Department of Basic and Clinical Oral Sciences, Umm Al-Qura University, Makkah, SAU

**Keywords:** patient education, youtube, social media, oral manifestations, covid-19

## Abstract

Objectives

This study aims to assess the quality of the most viewed videos on the YouTube website describing the oral manifestations of COVID-19 and appraise the medical information in the content.

Materials and methods

The top 200 most-viewed videos on YouTube using the keywords "COVID-19 oral manifestation," "oral symptoms of COVID-19," "oral lesions of COVID-19," "coronavirus and oral findings," and "dental manifestation of COVID-19" were analyzed. Two independent reviewers classified the English-language videos as useful, misleading, or personal views and identified the source of the videos. Reliability was calculated on a 5-point scale adapted from the DISCERN tool. The global quality scale (GQS) was used to determine the quality of the videos. In addition, the completeness of the information regarding the clinical presentation, pathogenesis, diagnostic tests, and treatment of COVID-19 oral manifestations was evaluated.

Results

After excluding non-English and irrelevant videos, 55 videos were analyzed. Thirty-two videos were classified as useful. Independent users uploaded the most videos (19, 51%). The mean reliability and GQS scores for useful videos were (3.24+1.4) and (2+0.75), respectively. The GQS score was significantly related to the reliability score (p<0.01). Videos scoring high in GQS also show high-reliability scores. In addition, videos with high GQS scores showed more comprehensive content, scoring >9 in the content aspect. The COVID tongue was the most discussed topic, followed by oral ulcers and oral mucormycosis.

Conclusions

Most of the YouTube videos were useful and had moderate quality. However, they show low reliability and lack comprehensive medical information on the topic. Healthcare providers should play a more active role in the educational information given on social media (SM) during global disease outbreaks.

## Introduction

The World Health Organization (WHO) considered the coronavirus disease (COVID-19) outbreak a public health emergency of international concern [1]. To date, more than 600 million cases have been reported worldwide [1]. COVID-19 can present with a range of clinical presentations, from asymptomatic cases to patients with mild symptoms such as fever, cough, and taste/smell loss. Severe symptoms, such as dyspnea or thromboembolic events, can manifest, especially in patients with comorbidities [2]. Literature suggests that oral ulcers could be an inaugural symptom of COVID-19 [3]. Other reported oral manifestations include geographic tongue, xerostomia, recurrent herpesvirus infection, candidiasis, necrotizing gingivitis, erythema multiforme‐like lesions, and salivary gland infections [4-6]. Most of the oral signs disappear as the disease resolves. However, they can cause significant disturbance to the patients [7].

The Internet provides easy access to medical information [8]. Seventy-four percent of patients searching social media (SM) for information about a disease, symptoms, or treatment searched for oral health-related information [9]. Health-related content on social media is constantly increasing and is freely accessed by the public; however, no standard system is available to ensure publication quality [10]. As a result, many posts contain unreliable, misleading, or outdated information. In addition, the content is usually posted by individual users, and opinions can be presented as facts without peer review. Most studies highlight the quality defects and low reliability of web-based health information [10,11], which may lead to the spread of false, inaccurate information.

YouTube is a platform that allows uploading, sharing, viewing, and commenting on videos. In 2023, it ranked second as the most popular social network worldwide, with 2514 million monthly users [12]. In fact, the top 100 YouTube videos on COVID-19 as of May 2020 generated over 245 million views [13].

Previous studies have assessed the medical information in YouTube videos on COVID-19 [13-15]. Dental information like infection control measures and dental emergencies during the pandemic was also reported [16]. However, to the best of our knowledge, COVID-19 oral manifestation content has not been addressed before. The main objective of this study was to evaluate and assess the most viewed videos on the YouTube website about the oral manifestations of COVID-19 and appraise the medical information in the content for both the public and healthcare providers.

## Materials and methods

A descriptive study to evaluate the quality of videos discussing oral symptoms of COVID-19 infection. On November 27, 2022, the YouTube website (www.youtube.com) was searched using the keywords "COVID-19 oral manifestation," "oral symptoms of COVID-19," "oral lesions of COVID-19," "coronavirus and oral findings," and "dental manifestation of COVID-19. The top 200 videos ranked by relevance (the YouTube default option, which uses an algorithm based on view count, upload date, rating, comments, bookmarks, and age of the user) were reviewed. The videos were saved in a file for future analysis, as YouTube search results are constantly changing. All videos about oral health, oral hygiene, dentistry, infection control, vaccines, and COVID-19 oral treatment were excluded. Also, videos in languages other than English, those without audio, and duplicate videos were excluded. All videos were viewed and analyzed for content by two independent dental specialists (a periodontist and an oral medicine specialist). This sample selection method has been used in similar studies in the literature [16-17].

Videos' basic information, such as country of origin, length of the video, and time since upload was recorded. Video popularity was used to assess audience interaction (defined as views per day, calculated as total views for a video divided by the number of days on YouTube) and video likability (number of "likes" for a video). The videos were classified based on their usefulness into three categories: (1) useful - if the video contained scientifically correct and accurate information about any aspect of the disease; (2) misleading - if the video contained scientifically unproven or inaccurate information based on currently available scientific evidence; and (3) patient views - if the video describes a patient’s personal experience while having an oral manifestation of COVID-19 [17].

The source of the videos was classified into five groups: (1) government/news agencies; (2) university channels/professional organizations; (3) health information websites (e.g., WHO); (4) independent health care providers (IHCP: dentists and medical doctors); and (5) independent non-healthcare providers (educational groups that were not identified as health care providers).

Assessment of reliability and completeness

All videos were analyzed for the completeness and reliability of the information. Reliability was calculated based on five yes or no questions (adapted from the DISCERN tool for assessment of written health information) [18]. The questions are: Are the aims clear and achieved? Are reliable sources of information used? (Publication cited, the speaker is a board-certified dental specialist); Is the information presented balanced and unbiased? Are additional sources of information listed for patient reference? Are areas of uncertainty/controversy mentioned?

One point is accredited for each question answered by yes, and 0 points for No. The score ranges from 0-5, with 5 indicating higher reliability, 

Completeness of information was scored on different aspects of disease information covered in the video (clinical presentation, pathogenesis, diagnostic tests, and treatment). Each aspect was evaluated based on a 3-point scale as 1: information was not discussed at all. 2: partial (incomplete) discussion of the information, 3: comprehensive discussion of the information. Videos were considered comprehensive when the total score is 10-12, moderately complete with a score of 7-9, and incomplete when the score was 4-6.

Quality assessment

The video quality was assessed using the modified global quality scale (GQS), which uses 4 points scale. 1: Poor quality and poor flow, incomplete information listed, many important topics missing, very limited value. 2: Moderate quality, suboptimal flow; some important information is adequately discussed, but others poorly discussed, somewhat useful. 3: Good quality and generally has a good flow. Most of the relevant information is listed, but some topics are not covered, useful for the audience.4: Excellent quality and flow, very useful for the audience.

The two investigators independently evaluated all the videos, and inter-rater consistency was determined using the Kappa coefficient. When inconsistency was identified, those were evaluated, and a consensus was reached on the final score.

Statistical analysis 

Data were analyzed using the Statistical Package for the Social Sciences version 20.0 software (SPSS Inc., Chicago, IL, USA). Descriptive data were stated as a number, percentage, mean, and standard deviation. Continuous variables were compared using an independent sample t-test, and nominal variables were compared by Chi-square test. P-value < 0.05 was considered significant.

## Results

**Figure 1:**
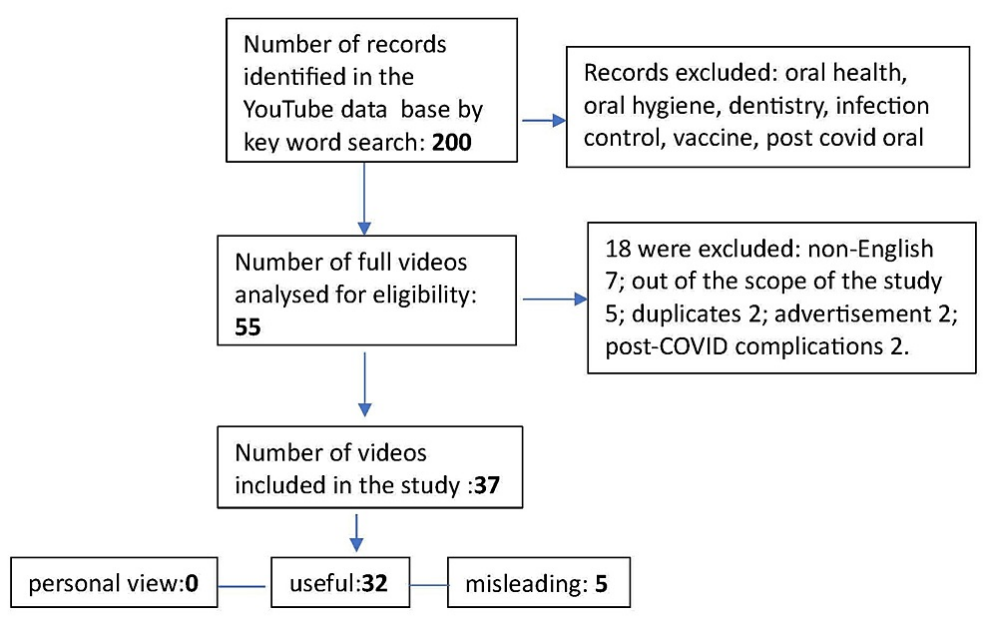
Flow diagram for video selection. The total number of YouTube videos screened and selected for evaluation.

The top 200 videos were screened, and 55 met the initial inclusion criteria. Of the 55 videos, 18 were excluded for the following reasons: seven videos were presented in non-English or mixed languages. In addition, five videos were out of the scope of this study. Two were duplicates, two were advertisement videos, and two were focused on post-COVID-19 complications. A total of 37 were considered for analysis in this study (Figure 1).

The mean duration of the videos was 17.5 minutes (range, 1-139 minutes). The mean view count was 98663.3 (range 2-1359557), and the mean days since upload were 658.40 (range 183-966 days). Thirty-two (86.4%) videos were classified as useful, and five (13.5%) were classified as misleading. None of the videos presented the patient's views. The Kappa score for the interrater agreement was 0.86.

Videos characteristic

The mean video lengths for useful and misleading videos were 19.23 ± 33 minutes and 3.28 ± 0.09 minutes, respectively (P<0.05). The average time since upload to YouTube was 658.1 ± 155.5 days for useful videos and 615.4 ± 227.4 for misleading ones (P>0.05). Useful videos showed higher popularity (176.06+500.24) and likability (770.03+2156.75) in comparison to misleading videos (3.74 ± 8.0 and 43.8 ± 91.25), respectively, and the difference was statistically significant (P<0.05) (Table 1). Total viewership and mean popularity were higher for the government/news agency source (71.9%, 671.9 ± 949.2) compared to health information websites (20.9%, 242.9 ± 523.4), while IHCP has a viewership percentage of 6% and a mean popularity of 24.5 ± 4209. Details on the video’s characteristics are presented in Table 1.

**Table 1:** Characteristics of videos on YouTube on oral manifestation of COVID-19. *Statistical significance P<0.05.

	Useful videos	Misleading videos	P-value
Number (%)	32 (86.4)	5(13.5)	
Total viewership	363,6115	14,429	0.01*
Total length, h:m:s	6:30:30	0:17:24	0.02*
Mean video length, min (SD)	19:23(33.75)	3:28(0.09)	0.04*
Mean duration on YouTube, days (SD)	658.1875(155.52)	615.4(227.49)	0.83
Mean popularity (SD)	176.06(500.24)	3.74(8.06)	0.002*
Mean “likes” (SD)	770.03(2156.75)	43.8 (91.25)	0.01*
Mean reliability (SD)	3.24 (1.4)	0.25(0.5)	0.0008*
Mean content (SD)	7.06(2.02)	4(0)	0.0008*
Mean global quality scale score (SD)	2(0.75)	1(0)	0.007*

**Figure 2:**
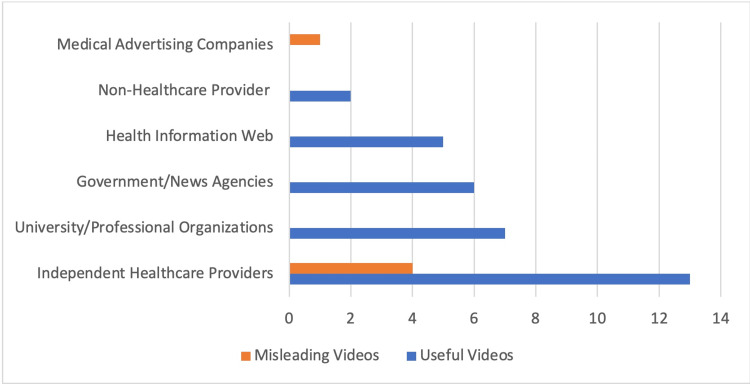
The source of the useful videos compared to misleading ones.

For useful videos, the most frequent source of videos was from independent health care providers with a total of 13 videos (35.1%), followed by university channels/professional organizations with seven videos (18.9%), government/news agencies with six videos (16.2%), health information websites with five videos (13.5%), and independent non-healthcare providers with two videos (5.4%). No videos were from medical advertising companies. The source of the misleading videos was one from a medical advertisement company and four from IHCP (Figure 2).

Country of origin and target audience

**Figure 3:**
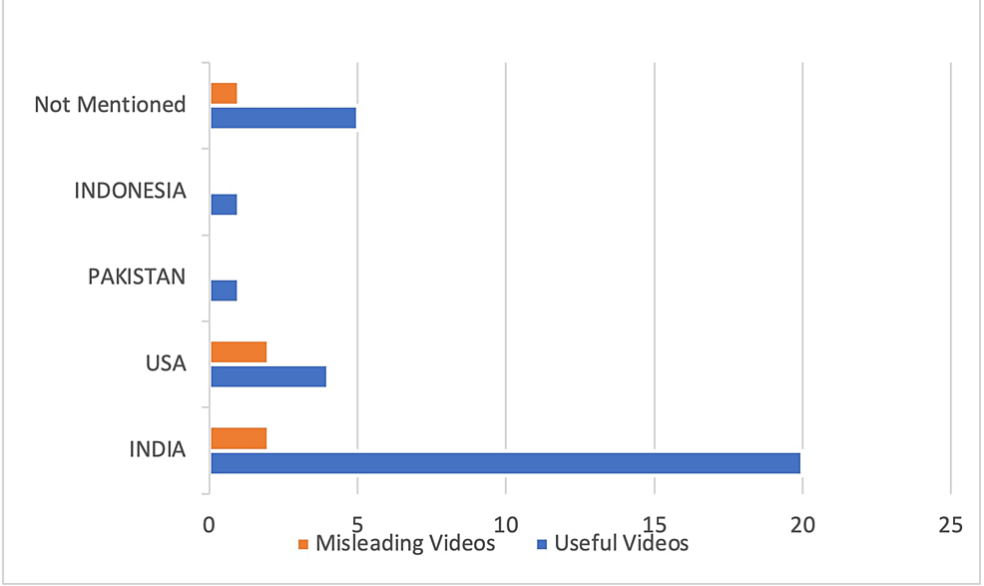
The country of origin of useful and misleading videos.

Twenty (54%) useful videos were from India; four (10.8%) were from the USA; one (2.7%) was from Pakistan; one (2.7%) was from Indonesia; and five (13.5%) did not mention the country of origin. On the other hand, 2 (5.4%) misleading videos were from the USA, and two (5.4%) were from India, and the country of origin of one of them was not available (Figure 3).

Six (16.2%) videos were directed towards dentists and HCPs and were not at the level of the general population. Five of them were useful, and one was a misleading video.

Reliability and contents

The mean reliability score for the useful videos was 3.24 ± 1.0. Twenty-three (71.8%) videos used reliable sources of information, but only 12 (37.5%) provided references to additional sources. However, in 22 (69%) videos, the information was presented balanced and unbiasedly.

The mean content score of the useful videos was 7.06 ± 2.12. Only four (12.5%) videos were considered comprehensive (score >10). On the other hand, 14 (43.75%) videos were considered deficient (scores 4-6). The remaining 14 videos scored between 7 and 9, making them moderately complete.

Most videos focused on the clinical presentation and signs of oral COVID-19 lesions. Investigations and treatment recommendations were not mentioned in most videos (22 [69%] and 25 [78%]), respectively. Oral ulcers, gingivitis, and periodontitis are the top symptoms reviewed. Eight videos discussed COVID tongue exclusively, and three addressed oral mucormycosis presentation and management.

The quality of the useful videos according to the source of information is presented in Table 2. Health information websites scored high in reliability and content, although a statistical difference was not achieved. Useful videos had statistically higher reliability and content scores compared to misleading videos (Table 1).

**Table 2:** Quality of useful videos (n = 32) on oral manifestation of COVID-19 according to the source of information. *Statistical Significance P<0.05.

	Total videos N=32	Government/news agency N=6	University/professional N=7	Health websites N=5	Independent - HCP N=13	Independent - non-HCP N= 2	P-value
Reliability of useful video (SD)	3.24(1)	2.3(1.4)	3.7(0.83)	3.8(0.84)	3.2(1.5)	3(2.8)	0.26
Are the aims clear and achieved (%)	28(87.5)	3 (50)	6(85.7)	5(100)	12(92.3)	2(100)	0.17
Are reliable sources of information used (%)	23(72)	5(83.3)	5(71.4)	4(80)	8(61.5)	1(50)	0.88
Is the information presented balanced and unbiased (%)	22(69)	2(25)	6(85.7)	4(80)	9(69.2)	1(50	1.99
Are additional sources of information listed for patient reference (%)	12(37.5)	0	5(71.4)	2(40)	4(31)	1(50)	0.2
Are areas of uncertainty mentioned (%)	22(69)	4(67)	4(57)	4(80)	9(69.2)	1(50)	0.68
Content score (SD)	7.06(2.12)	5(0)	7.3(2.3)	7.6(1.1)	7.8(2.01)	7 2.8)	0.05
Clinical Presentation (SD)	2.4	2(0)	2.14(0.89)	2.8(0.44)	2.5(0.66)	2.5(0.7)	0.13
Pathogenesis (SD)	1.9	1 (0)	1.9(0.89)	2.6(0.89)	2.2(0.98)	1.2(0)	0.02*
Investigations (SD)	1.42	1(0)	1.6(0.84)	1.2(0.44)	1.6 (0.77)	1.4 (0.7)	0.23
Treatment (SD)	1.4	1(0)	1.7(0.95)	1(0)	1.4(0.65)	1.4(1.4)	0.14
Mean global quality scale score (SD)	2(0.75)	1.33(0.51)	2.14(0.69)	2.2(0.44)	2.2(0.83)	1.5(0.70)	0.08
Low GQS (%)	8(25)	4(66.6)	1(14.3)	0	2(15.4)	1(50)	
Moderate GQS (%)	18(56.2)	2(33.3)	4(57.1)	4(80)	7(53.8)	1(50)	
High quality GQS (%)	7(22)	0	2(28.6)	1(20)	4(30.8)	0	

Global quality scale

The mean GQS for useful videos was 2 ± 75. The quality of most videos (18, 56%) was moderate. Only six videos had good quality (score =4), and one video only had an excellent score. There was no difference in popularity between videos with high GQS (3-4) (n = 7) and low GQs (1-2) (n = 26), with the mean video popularity for high GQS being 180.1 ± 441.7 compared to the mean popularity of low GQS of 167.6 ± 510.4 (P = 0.46). The GQS score was significantly related to the reliability score (p < 0.01), with videos scoring high in the GQS also showing a high-reliability score. In addition, videos with high GQS scores showed more comprehensive content, scoring >9 in the content aspect.

## Discussion

This study demonstrates that most YouTube videos about the oral manifestations of COVID-19 infection are useful. IHCPs were the most common source of information on the oral manifestation of COVID-19, followed by academic institutes. The popularity and likability were higher for useful videos compared to misleading ones. However, most videos lacked comprehensiveness, particularly in the investigation and treatment aspects. The videos, in general, showed moderate quality scores. These findings are consistent with prior studies evaluating YouTube videos as a source of medical information [15,17,19].

None of the videos were from the CDC or WHO, although they are quoted as sources of information in many videos. In addition, only seven videos were from academic institutions. In general, those videos were recordings of webinars or academic lectures and were therefore directed to practising dentists rather than the general population. A similar finding was reported by D’Souza et al. [15] when evaluating medical information about COVID-19 on YouTube. Therefore, academic and reputable centres should recognize the opportunities provided by YouTube (and other SM platforms) to disseminate accurate health-related information that can reach communities at a relatively low cost [20]. If credible medical organizations fail to utilize SM platforms to disseminate validated information, then the chances of misleading information spreading increase.

In the study, the global quality score of useful videos was significantly related to the reliability score assessed by the DISCERN tool, as in similar previous studies [11,19] indicating that the content and the quality of the included videos were interrelated. In terms of comprehensiveness, most of the useful videos scored higher in clinical presentation and pathogenesis than in investigation and treatment. Similarly, previous studies evaluating YouTube medical content on COVID-19 indicated that videos showed more comprehensive information on clinical symptoms of COVID-19 rather than diagnosis and treatment [15]. This may be due to the fact that during the pandemic, there was more emphasis on finding new cases and possible clinical presentations than on treating them.

The study included a limited number of videos. However, several practical implications can be inferred from the analysis to help improve educational outcomes. First, it is essential to identify the targeted audience of the video, and this should be stated at the beginning of the video or in the description. Many videos are intended to inform medical professionals and include medical information that can be of little value to the general patient population. Moreover, many videos had photos of clinical presentations or surgical procedures that can be disturbing to nonmedical personnel. The identification of such content should be evident in the video description. Also, we noticed that many videos intended for the public contained medical terminologies that might not be familiar to the layperson and were not explained during the video. A recent systematic review found that most health websites exceed the recommended readability levels intended for the public and, therefore, may hinder the general population from being adequately informed [10]. Although studies have yet to address the recommended level for the spoken language, one can infer that medical terminology without explanation can affect the intended educational outcome of the post.

Second, to ensure the creditability of the source, the presenter or the video creator should be identified at the beginning of the video, and their professional qualifications should be clearly indicated. Literature shows that the most trusted sources of online health information from the patient's perspective included medical doctors, medical universities, and government sources [21]. As in many similar studies, independent users were the source of most videos [13]. Unfortunately, in many videos, the professional identity of the presenters/uploaders was not easily detectable.

In addition, it is essential to add references or a supplementary source list at the end of the video. It is also crucial to acknowledge the need for continuous updates as information on the COVID-19 outbreak is changing by the minute.

The COVID tongue was one of the topics discussed extensively in the videos, followed by oral ulcers and gingivitis. Interestingly, oral mucormycosis is commonly addressed in the videos, which originated in India. The high surge in cases of COVID-19 in India during the second wave of the pandemic was associated with increased reporting of invasive mucormycosis [22]. The estimated prevalence of mucormycosis in India is about 70 times higher than in global data [23]. Thus, oral mucormycosis is a common complication affecting the medically complex population in that part of the world.

Limitation and strength

The study was limited to analyzing videos in the English language only and excluded videos in other languages. We were also limited to direct YouTube searches, and we did not include videos linked/embedded in other websites. The study did not include post-COVID or long COVID symptoms or the effects of vaccination. The DISCERN tool used for reliability assessment is a general tool and lacks many of the criteria that are important for assessing specific information content. It was originally designed to be used for written content. However, it was adopted for online content and used by several studies [13,15,17,19].

The strength of this project is that it would involve videos that were analyzed by specialists with evidence-based knowledge of the topic, and public evaluation of the videos is needed. The study used objective criteria to classify videos as useful, misleading, or personal views, as well as reliability and content quality evaluations done using established rating scales that have been used in similar studies [13,17,19].

## Conclusions

YouTube is a popular, open-access video-sharing website that hosts a high number of videos on different aspects of many diseases. In this study, we evaluated the most viewed videos on the YouTube website discussing oral manifestations of COVID-19. Although most of the reviewed videos were classified as useful, they still lack comprehensive medical information on the topic. It is recommended that reputable organizations provide accurate and reliable medical information to be available for internet users, and healthcare providers play an active role in uploading educational information on SM, especially YouTube, during global disease outbreaks.

## References

[1] (2022). Coronavirus disease (COVID-19). https://www.who.int/health-topics/coronavirus#tab=tab_3.

[2] Jutzeler CR, Bourguignon L, Weis CV (2020). Comorbidities, clinical signs and symptoms, laboratory findings, imaging features, treatment strategies, and outcomes in adult and pediatric patients with COVID-19: A systematic review and meta-analysis. Travel Med Infect Dis.

[3] Chaux-Bodard AG, Deneuve S, Desoutter A (2020). Oral manifestation of Covid-19 as an inaugural symptom?. J Oral Med Oral Surg.

[4] Brandini DA, Takamiya AS, Thakkar P, Schaller S, Rahat R, Naqvi AR (2021). Covid-19 and oral diseases: crosstalk, synergy or association?. Rev Med Virol.

[5] Farid H, Khan M, Jamal S, Ghafoor R (2022). Oral manifestations of Covid-19-A literature review. Rev Med Virol.

[6] Eghbali Zarch R, Hosseinzadeh P (2021). COVID-19 from the perspective of dentists: a case report and brief review of more than 170 cases. Dermatol Ther.

[7] Qu X, Zhou XD (2020). [Psychological intervention for patients with oral disease during the pandemic period of COVID-19]. Zhonghua Kou Qiang Yi Xue Za Zhi.

[8] Vance K, Howe W, Dellavalle RP (2009). Social internet sites as a source of public health information. Dermatol Clin.

[9] Almaiman S, Bahkali S, Alabdulatif N, Bahkaly A, Al-Surimi K, Househ M (2016). Promoting oral health using social sedia platforms: seeking arabic online oral health related information (OHRI). Stud Health Technol Inform.

[10] Stern J, Georgsson S, Carlsson T (2022). Quality of web-based information about the coronavirus disease 2019: a rapid systematic review of infodemiology studies published during the first year of the pandemic. BMC Public Health.

[11] Yüce MÖ, Adalı E, Kanmaz B (2021). An analysis of YouTube videos as educational resources for dental practitioners to prevent the spread of COVID-19. Ir J Med Sci.

[12] (2023). Most popular websites worldwide as of November 2022, by total visits. https://www.statista.com/statistics/1201880/most-visited-websites-worldwide/.

[13] Andika R, Kao CT, Williams C, Lee YJ, Al-Battah H, Alweis R (2021). YouTube as a source of information on the COVID-19 pandemic. J Community Hosp Intern Med Perspect.

[14] Szmuda T, Syed MT, Singh A, Ali S, Özdemir C, Słoniewski P (2020). YouTube as a source of patient information for Coronavirus disease (COVID-19): A content-quality and audience engagement analysis. Rev Med Virol.

[15] D'Souza RS, D'Souza S, Strand N, Anderson A, Vogt MN, Olatoye O (2020). YouTube as a source of medical information on the novel coronavirus 2019 disease (COVID-19) pandemic. Glob Public Health.

[16] Ozdede M, Peker I (2020). Analysis of dentistry YouTube videos related to COVID-19. Braz Dent J.

[17] Singh AG, Singh S, Singh PP (2012). YouTube for information on rheumatoid arthritis--a wakeup call?. J Rheumatol.

[18] Charnock D, Shepperd S, Needham G, Gann R (1999). DISCERN: an instrument for judging the quality of written consumer health information on treatment choices. J Epidemiol Community Health.

[19] Özkan E, Acar AH (2022). YouTube™ as a source of information for patients regarding dental implant failure: a content analysis. J Craniofac Surg.

[20] Farsi D (2021). Social media and health care, part I: literature review of social media use by health care providers. J Med Internet Res.

[21] Dutta-Bergman M (2003). Trusted online sources of health information: differences in demographics, health beliefs, and health-information orientation. J Med Internet Res.

[22] Al-Tawfiq JA, Alhumaid S, Alshukairi AN (2021). COVID-19 and mucormycosis superinfection: the perfect storm. Infection.

[23] Prakash H, Chakrabarti A (2021). Epidemiology of mucormycosis in India. Microorganisms.

